# Development and psychometric evaluation of the emergency nurses’ professional resilience tool

**DOI:** 10.1371/journal.pone.0269539

**Published:** 2022-06-07

**Authors:** Roohangiz Norouzinia, Mohammad Hosein Yarmohammadian, Masoud Ferdosi, Gholamreza Masoumi, Abbas Ebadi

**Affiliations:** 1 Social Determinants of Health Research Center, Alborz university of Medical Sciences, Karaj, Iran; 2 Health Management and Economics Research Centre, Isfahan university of Medical Sciences, Isfahan, Iran; 3 Emergency Management Research Centre, Iran University of Medical Sciences, Tehran, Iran; 4 Behavioural Sciences Research Centre, Life style Institute, Baqiyatallah University of Medical Sciences, Tehran, Iran; 5 Nursing Faculty, Baqiyatallah University of Medical Sciences, Tehran, Iran; Yamaguchi University: Yamaguchi Daigaku, JAPAN

## Abstract

**Background:**

There is no specific tool for measuring the professional resilience of emergency nurses. Therefore, the present study aimed to design and psychometrically evaluate a new tool named the emergency nurses’ professional resilience tool.

**Method:**

This mixed-method sequential exploratory study was conducted in two phases: (1) item generation using literature review and evaluation of the results of a qualitative study and (2) psychometric evaluation of the developed scale. The face, content, and construct validity (exploratory and confirmatory factor analysis), reliability (internal consistency, relative, and absolute), and accountability were assessed in the population of Iranian nurses (N = 465) during March 2019-June 2020.

**Results:**

The tool designed for assessing the professional resilience of Iranian nurses included 37 items. The average scale content validity index (S-CVI/Ave) was equal to 0.94. The exploratory factor analysis revealed five factors, including professional competencies, emotional-cognitive characteristics, external support, in addition to behavioral and cognitive strategies, and explained 75.59% of the whole variance. Cronbach’s alpha and intraclass correlation were 0.915 and 0.888, respectively. Construct validity for five factors was established with acceptable model fit indices [Chi–square/df = 1336.56/619, p < .001]; [Comparative Fit Index [CFI] = 0.96]; [Non-Normed Fit Index [NNFI] = 0.96]; [Root Mean Square Error of Approximation (RMSEA) = 0.074 and 90 Percent Confidence Interval = (0.069; 0.080)]; and [SRMR = 0.095].

**Conclusions:**

According to the findings of the current study, the emergency nurses’ professional resilience tool can be used by healthcare managers as a valid and reliable scale to evaluate the professional resilience of nurses to designate them as nurses working in emergency and disaster situations.

## Introduction

The emergency department is known as a stressful workplace on which there is almost a global consensus. When an incident happens, the emergency department and emergency nurses are always the first group in the hospitals to deal with many casualties. Therefore, nurses are the largest group of healthcare providers, especially while responding to emergencies events [1, 2].

The results of an integrative review indicated that the emergency department employees were exposed to more stress than employees in other departments [3]. According to a study by Frankenberger (2014), these work-related severe stressors can adversely affect the mental health of nurses, which in turn influences patient care [4]. The nurses caring for traumatized patients may experience severe and traumatic emotional reactions that can lead to compassionate fatigue if unrecognized and improperly treated [5]. Moreover, it has been indicated that some characteristics, such as skill level, autonomy, work experience, and teamwork, reduced the effects of stress [3].

Resilience is an approach for responding to stress and problems and means jumping forward or rising again [6]. Resilience in the workplace has been defined as the ability to maintain personal and professional health while facing professional stress and difficulties [7], increasing clinical service quality [8]. Factors affecting nurses’ resilience are work-life balance, hope, control, support, professional identity, and clinical supervision [7]. From the researchers’ point of view, resilience is a necessary component for success in nursing because the working conditions would be very difficult in the absence of resilience [9, 10].

Several scales evaluated workplace resilience, each with a different concept and developed for different cultural backgrounds. Due to the different concepts of resilience, there is no unified, reliable, and valid tool that can be used in all professional groups and communities. Therefore, it is recommended that more specific and multifactor measures be developed based on each group’s cultural background, profession, and available resources [11].

The present study aimed to design and evaluate the psychometric characteristics of a specific tool for assessing the emergency nurses’ professional resilience. The tool considered various personal and professional dimensions, external support, and self-regulatory processes by which nursing managers could evaluate the professional resilience of their nurses and plan for it.

## Methods

### Study design

This mixed-method sequential exploratory study was conducted in two phases: (1) item generation based on literature review and the results of a qualitative study and (2) psychometric evaluation of the developed scale during March 2019- June 2020.

At the first stage, the qualitative content analysis aimed to explain the emergency nurses’ professional resilience. The operational definition of the construct and its dimensions, along with reviewing the literature and tools related to the concept of resilience, led to the production of a pool of items. At the second stage, the psychometric properties of the tool were investigated.

### Item generation phase

In this phase, items were generated according to the literature and the findings of a qualitative study. Firstly, a systematic review of papers published from 1989 until August 1, 2019, concerning workplace resilience tools was conducted at PubMed, Web of Science, SCOPUS, and Google Scholar databases. The keywords included resilience, instruments, tool, inventory, questionnaire, scale, workplace, staff, and occupational. In addition, a general search was performed in the google search engine for “resilience instrument”. A total of 11 articles were selected in the study after reviewing 3057 extracted articles [11]. Finally, several items of two measures were utilized in the initial item pool [12, 13].

In the second step, 21 semi-structured individual interviews of 15–45 minutes were organized to understand better the resilience concept among nurses working in the trauma emergency department of two trauma centers in two northwest and central provinces of Iran. The interviews were recorded and transcribed. The texts of the interviews were analyzed using the deductive content analysis approach proposed by Elo and Kingas [14], with a directed content analysis method using MAXQDA software version 10 [15].

### Item reduction

The emergency nurses’ professional resilience components extracted in the first and second stages of item generation have emerged. Based on the extracted codes, categories, and subcategories, a pool of 83 items was obtained. The research team members reviewed and edited the extracted items at two stages, and some were removed and modified. Finally, the preliminary format of the emergency nurses’ professional resilience scale with 44 items was made. Only the items extracted from the qualitative study remained in this version of the scale. The response options were based on a five-point Likert scale with always, often, sometimes, rarely, and never answers.

### Face validity

The face validity of the questionnaire was examined using qualitative and quantitative methods [16]. To evaluate qualitative face validity, ten nurses in the trauma emergency department were asked to comment on the appropriateness, difficulty, and ambiguity of the items. To assess quantitative face validity, 15 trauma emergency nurses were asked to determine the importance of the items on a Likert-type scale from quite important (score 5) to not important (score 1). To calculate the impact factor of the items, the formula of frequency × importance was used. Items with an impact factor higher than 1.5 were considered appropriate [17]. Participants in this step were 28–42 years old (36.4±3.87) and had more than two years (5.4±2.33) of experience working in the emergency department.

### Content validity

Content validity was assessed using qualitative and quantitative methods. At this step, 15 experienced faculty members with a background in instrument development (N = 2), nursing (N = 6), psychology (N = 4), and health in emergencies and disasters (N = 3) from different universities were asked to assess and comment on the wording, item allocation, and scaling of the items. At this step, two items were merged and the items of tool were reduced to 43 items.

Furthermore, the content validity ratio (CVR) and content validity index (CVI) were calculated. To report CVR, ten experts in instrument development (N = 2), nursing (N = 3), psychology (N = 3), and health in emergencies and disasters (N = 2) were asked to score each item on a three-point scale as ‘necessary’, ‘useful but not necessary’, and ‘unnecessary’. Based on the Lawshe Table, the items with the CVR of 0.62 and higher were preserved [18].

To calculate the CVI, the same ten experts were requested to rate the relevance of each item on a 4-point Likert scale. To assess item-level CVI (I-CVI), the number of experts who scored a particular item as 3 or 4 was divided by the total number of experts. A CVI value of 0.78 or higher was considered satisfactory. The S-CVI average (S-CVI/Ave) technique was applied to calculate the scale-level CVI (S-CVI). An S-CVI/Ave of 0.9 and higher were considered very good content validity [19]. Three items were removed based on the above considerations due to CVR and CVI of lower than 0.62 and 0.78, respectively. As a result, the items of NPRT were reduced to 40 items.

### Construct validity

Exploratory and confirmatory factor analyses were used to determine to construct validity. According to the normal distribution of variables with the skewness of ±3 and kurtosis of ±7, the maximum likelihood exploratory factor analysis (EFA) [20] was used to extract hidden factors, followed by a Promax rotation with SPSS 22 (SPSS Inc., Chicago, IL, USA). A total of 254 emergency nurses were included in the study through convenience sampling. The inclusion criteria were working in the trauma emergency department, having at least 2 years of experience in the emergency department, and the lack of severe physical and mental problems. The sample size was considered 5–10 samples for each item designed in the tool [21]. The electronic version of the questionnaire consisting of demographic questions and 40 items was completed by the emergency nurses. Social media, namely WhatsApp and Telegram, were used for data collection. The Kaiser-Meyer-Olkin test (KMO>0.7) to evaluate the adequacy of sampling and Bartlett’s test of sphericity were applied to examine the correlation matrix between items (P<0.05) [21]. The eigenvalues, at least three items, and 5% variance in each factor were used to determine the number of factors. The items with a factor loading value of 0.3 or more were considered appropriate, and the eigenvalue of 1 or less was ignored [22]. Furthermore, correlations of sub-factors were examined.

In the second step, confirmatory factor analysis (CFA) and the most common goodness-of-fit (GFI) indices according to the accepted threshold were estimated by LISREL software 8.8 to evaluate the extracted factors from the qualitative study. To determine fit the model, the following criteria were measured: the normed fit index (NFI>0.9 acceptable), non-normed fit index (NNFI), comparative fit index (CFI>0.9 acceptable), GFI (>0.9 acceptable), Chi-square per degree of freedom (χ^2^/df<3), root mean square error of approximation (RMSEA<0.08) [23], and standardized root mean square residual (SRMR<0.08 acceptable) [24]. According to researchers, the minimum sample size required for CFA was 200 [23, 25]. This step was conducted on 212 emergency nurses different from the previous sample and selected using the convenience sampling method.

### Reliability

The reliability of the scale was assessed utilizing internal consistency and stability [26]. Cronbach’s alpha (≥0.7) was calculated to examine the internal consistency of the scale [27]. The stability of the scale was evaluated using the test-retest method and intraclass correlation coefficient (ICC>0.8 was acceptable) [28]. Therefore, a small sample of emergency nurses (N = 30) completed the NPRT twice with an interval of 2 weeks. The standard error of measurement (SEM) was examined for the absolute reliability analysis. The less the amount of SEM, the more the reliability [29]. The SEM has two types of SEM agreement and SEM consistency. In the present study, SEM agreement was measured using the following formula: SEM agreement = SEM = SD√(1-ICC) [30].

### Repeatability

Stability and agreement are called repeatability. The agreement is positive if the smallest detectable change (SDC) is higher than the minimally important change (MIC). The SEM measurement has a clinical application and indicates the range of each score [30]. Interventions, such as passing a training course can increase resilience. Consequently, this construct can be considered a clinical case. Using SEM, the amounts of SDC were measured for each subscale and the whole scale. The SDC was considered with a reliability coefficient of 0.95. Therefore, the value with P<0.05 indicated the smallest detectable change.

### Ethical consideration

This study is the result of a doctoral dissertation approved by Regional Ethics Committee affiliated to Isfahan University of Medical Sciences, Isfahan, Iran (Ethics Code No: IR. MUI. RESEARCH. REC. 1398. 272). After explaining the aims of the study to the participants, they were informed that they could withdraw from the study at any time. The written informed consent form of the study was read and signed by participants. Moreover, the participants’ name was replaced with a code, and they were assured of the confidentiality of their names.

## Results

After integrating the items extracted from available and relevant workplace resilience scales and the qualitative phase, the preliminary tool containing 83 items was created. After reviewing and removing the duplicate items, 44 items remained. The evaluation of face validity revealed that none of the items had an impact factor of less than 1.5. In the qualitative review of content validity, all changes proposed by experts were applied. The most important alterations were changes in the structure and appearance of the items and merging both items. Finally, 43 items remained. In CVR evaluation, three items with a CVR of 0.4 were removed, and the number of items decreased to 40. The numerical values of the CVI of all items were greater than 0.79. Based on the mean scores of I-CVI, the S-CVI/Ave was equal to 0.94.

In the present study, 254 nurses working in the emergency department completed the electronic questionnaires. The mean age and work experience of the participants were 33.94±7.11 and 10.29±7.56 years, respectively. We observed that 61.8% of the participants were female, 61.8% were married, and 85.5% had bachelor’s degrees.

In the factor analysis model, the Kaiser-Meyer-Olkin (KMO) test value was 0.892, and Bartlett’s test value was 4494.218 (P<0.001). Five factors were extracted and named as “professional competencies” (14 items), “emotional-cognitive characteristics” (13 items), “behavioral strategies” (five items), “external support” (three items), and “cognitive strategies” (two items). These five factors had specific values of 10.665%, 2.778%, 2.38%, 1.995%, 1.601% respectively, and 75.59% of the total variance of variables of the emergency nurses’ professional resilience questionnaire explained. The Promax rotation was performed according to the scree plot and the total variance table. Three items were not loaded in any of the factors (factor loading<0.3), and only two items were included in factor five. Given that there are two items with the factor loadings of 0.92 and 0.87, it was maintained as a factor (Table 1).

**Table 1:** Exploratory factors extracted from the emergency nurses’ professional resilience questionnaire.

N of item	Item	Factor Name and Number	Factor loading	Percentage of variance	Initial Eigenvalues
**9**	I have enough experience to work in emergencies or difficult and complex situations during a crisis or medical emergencies.	**1** **Professional competencies**	0.841	22.74	10.665
**11**	I can control and manage a large number of casualties during a crisis or medical emergencies.		0.807		
**8**	I have enough knowledge to work in the emergency department during a crisis or medical emergencies.		0.798		
**10**	I can perform my duties quickly, accurately, and completely during a crisis or medical emergencies.		0.749		
**23**	I have great self-confidence in complex emergencies.		0.608		
**7**	I perform emergency procedures during a crisis or medical emergencies.		0.597		
**21**	I can make rational decisions during a crisis or medical emergencies.		0.480		
**2**	I try to stay calm during a crisis or medical emergencies.		0.477		
**20**	I can control my emotions during a crisis or medical emergencies.		0.464		
**16**	I can manage aggressive companions during a crisis or medical emergencies.		0.448		
**24**	I welcome difficult and complex situations.		0.414		
**15**	I have the ability to communicate well with patients during a crisis or medical emergencies.		0.389		
**12**	I know my professional abilities well during a crisis or medical emergencies.		0.362		
**1**	I endure the hardships of working in the emergency department well during a crisis or medical emergencies.		0.342		
**38**	I value patient service.	**2** **Emotional-cognitive characteristics**	0.836	21.95	2.778
**35**	I enjoy trying to save patients’ lives.		0.759		
**5**	I feel satisfied helping the sick and casualties during a crisis or medical emergencies.		0.698		
**40**	I consider nursing a valuable and meaningful profession.		0.691		
**6**	I see patients as members of my family during a crisis or medical emergencies.		0.603		
**39**	I pay more attention to the positive aspects of my profession.		0.592		
**3**	I do not spare any effort to save the patients’ lives during a crisis or medical emergencies.		0.551		
**13**	I work closely with other members of the care team during a crisis or medical emergencies.		0.537		
**37**	I agree that I should not be disappointed.		0.503		
**14**	I can communicate appropriately with the care team during a crisis or medical emergencies.		0.471		
**4**	I try to understand the patients’ suffering during a crisis or medical emergencies.		0.462		
**25**	I am looking to learn new necessary things for my profession.		0.362		
**27**	My family and friends understand my work situation and my feelings.		0.352		
**28**	I have personal plans and interests and I follow them. (sports, art activities, music, relaxation, and meditation techniques, study,. . .)	**3** **Behavioral strategies**	0.877	11.45	2.380
**29**	I spend time with my friends, family. and colleagues.		0.757		
**31**	I travel to reduce the stress of my workplace problems.		0.683		
**30**	I have religious and spiritual programs.		0.551		
**32**	I do not bring the problems of my workplace into the normal course of my life.		0.408		
**18**	I am sure that there is sufficient personal protective equipment to care for high-risk patients during a crisis or medical emergencies.	**4** **External support**	0.929	10.69	1.995
**19**	I am sure that there is sufficient manpower to replace and support during a crisis or medical emergencies.		0.670		
**17**	I am sure that there is sufficient medical equipment to care for patients during a crisis or medical emergencies.		0.645		
**34**	I have accepted that dealing with the patients’ traumatic injuries is an integral part of my job.	**5** **Cognitive strategies**	0.924	8.76	1.601
**33**	I have accepted that dealing with patients’ mortality is an integral part of my job.		0.871		

The results of correlations between sub-factors are shown in Table 2.

**Table 2:** Correlations among each sub-factor of the emergency nurses’ professional resilience questionnaire.

		Factor 1	Factor 2	Factor 3	Factor 4	Factor 5	Total
Factor 1	Pearson Correlation		.681**	.271**	.486**	.364**	.855**
	Sig. (2-tailed)		.000	.000	.000	.000	.000
Factor 2	Pearson Correlation			.386**	.656**	.326**	.910**
	Sig. (2-tailed)			.000	.000	.000	.000
Factor 3	Pearson Correlation				.305**	.177**	.564**
	Sig. (2-tailed)				.000	.005	.000
Factor 4	Pearson Correlation					.127*	.701**
	Sig. (2-tailed)					.044	.000
Factor 5	Pearson Correlation						.434**
	Sig. (2-tailed)						.000
Total	Pearson Correlation						
	Sig. (2-tailed)						

**. Correlation is significant at the 0.01 level (2-tailed).

*. Correlation is significant at the 0.05 level (2-tailed).

The CFA was performed on items with a single-factor model and 5-factors model. The model was not run with a single-factor, and the 5-factors model extracted from the qualitative study consistent with EFA was approved. The second sample analysis showed that the mean age and work experience of 211 nurses working in the emergency department who completed the electronic questionnaires were 34.46±8.45 and 10.68±7.53 years, respectively. Our results demonstrated that 71.7% of the participants were female, 52.8% were married, and 82.5% had bachelor’s degrees. In CFA, Chi-square GFI test results were obtained [χ^2^ = 1336.56, P<0.001]. Afterwards, other indices were evaluated to fit the model.

Based on an acceptable level of indices as CFI = 0.96, NNFI = 0.96, RMSEA = 0.074, 90% confidence interval = 0.069–0.08, and SRMR = 0.095, the appropriate fit of the final model was confirmed (Table 3).

**Table 3:** Fit indicators of confirmatory factor analysis model of assessment the emergency nurses’ professional resilience questionnaire.

Fit Indicators* Confirmatory factor analysis model	χ2	df	P-value	CMIN/DF	RMSEA	PNFI	AGFI	CFI	NFI	IFI	GFI	SRMR
First order after construction modification	**1336.56**	**619**	**<0.001**	**2.15**	**0.074**	**0.87**	**0.95**	**0.96**	**0.94**	**0.96**	**0.96**	**0.095**

*: Acceptable values of Index of PNFI (>0.5), AGFI (>0.8), GFI, CFI, IFI, NFI (>0.9), RMSEA (<0.08), CMIN/DF (3 <Good, 5<Acceptable), SRMR (<0.1)

**Fig 1:**
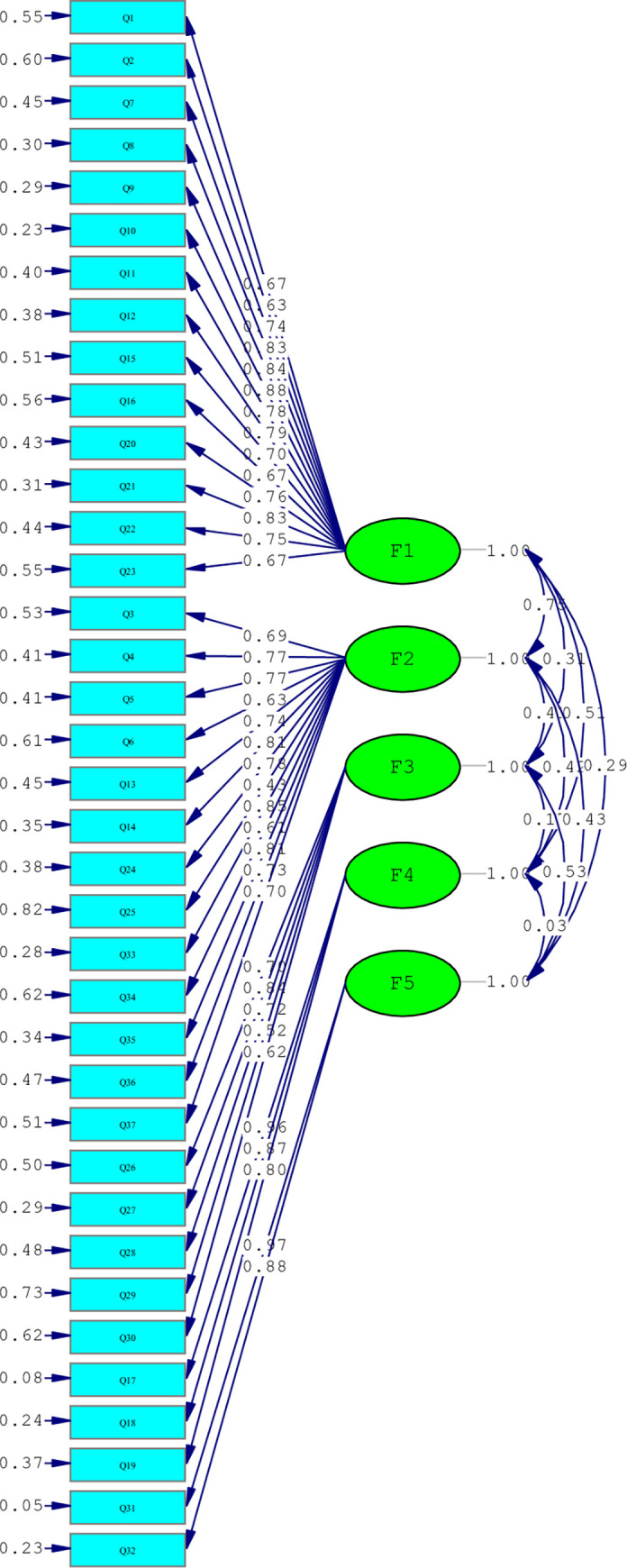
Constructions of evaluation of the emergency nurses’ professional resilience questionnaire: A modified confirmatory.

The final modified CFA model of the construction of the emergency nurses’ professional resilience is shown in Fig 1.

According to the results presented in Table 3, the internal consistency of the questionnaire based on the Cronbach’s alpha coefficient for the whole questionnaire was 0.915. moreover, ICC was found as 0.888 (95% confidence interval: 0.766–0.946). The SEM was obtained as 3.456 applying the formula. Therefore, it can be claimed that the scores obtained from the instrument in the range of about 3.5 had errors. The minimum and maximum instrument scores were 37 and 185, respectively. In addition, the mean scores of the test-retest were 155.6 and 154.3, respectively. Therefore, the error value of 3.5 seemed to be good. The SDC and MIC values were calculated to detect the agreement in the subscales and the total score of the instrument (Table 4).

**Table 4:** Internal consistency and repeatability (SEM, SDC, MIC and Agreement) of assessment the emergency nurses’ professional resilience questionnaire.

Index Factor	No. of items	Cronbach’s alpha Coefficient	ICC	CI, 95%	variation range	SEM	SDC	MIC	Agreement
				Low-Upper					
**1**	**14**	**0.859**	**0.770**	**0.522–0.890**	**46–70**	**1.761**	**4.878**	**0.543**	**+**
**2**	**13**	**0.869**	**0.752**	**0.488–0.881**	**49–65**	**1.282**	**3.553**	**0.067**	**+**
**3**	**5**	**0.771**	**0.778**	**0.540–0.894**	**11–25**	**1.099**	**3.046**	**0.309**	**+**
**4**	**3**	**0.833**	**0.782**	**0.540–0.897**	**6–15**	**0.645**	**2.873**	**0.054**	**+**
**5**	**2**	**0.911**	**0.682**	**0.325–0.850**	**7–10**	**0.300**	**0.831**	**0.044**	**+**
**Total tool**	**37**	**0.915**	**0.888**	**0.766–0.946**	**128–185**	**3.456**	**9.579**	**0.80**	**+**

## Discussion

This study was conducted to evaluate the psychometric properties of a new specific tool for assessing nurses’ professional resilience, NPRT, among nurses in emergency departments during March 2019-June 2020. The primary scale was designed after conducting a systematic review [11] and a qualitative study [15]. Content and face validity, as well as EFA, CFA, and Cronbach’s alpha coefficient, were used to determine internal consistency and reliability.

The emergency department has the potential to pose emotional challenges in nursing staff due to its unpredictable and stressful nature [31]. At times of patient overload, stress substantially increases in the emergency department nurses, resulting from limited patient care time, work overload, or psychosocial reasons [32]. Frankenberger (2014) demonstrated that emergency department nurses are exposed to some severe professional stressors with potentially negative effects on the nurses’ psychological health and, in turn, on the patients’ care [4]. The nurses in charge of traumatized patients may suffer severe and traumatic emotional responses that will lead to compassion fatigue if left unrecognized and mishandled [5]. Jackson et al. (2007) believed that nurses require resilience to succeed in their profession, as the work conditions can become quite difficult [33]. Professional resilience is a combination of characteristics, processes, and support systems that enables individuals to return to their previous functional status or health conditions after a traumatic incident at their workplace [12].

The EFA indicated that the scale has five factors, including “professional competencies” (14 items), “emotional-cognitive characteristics” (13 items), “behavioral strategies” (five items), “external support” (three items), and “cognitive strategies” (two items). According to our results, the variance of five factors is 75.59%, above 50%, and acceptable. All the factors of resilience were positively correlated with each other.

The first factor of NPRT was professional competencies. Even though resilience studies have been conducted in the workplace [34, 35], workplace resilience in the nursing profession is not yet fully understood [36], and the emphasis of nursing resilience research is often on individual resilience [37]. In the study by Cameron and Brownie [38], clinical proficiency was an essential determinant in resilience. Other researchers also found that managing job demands [39] effectively or business confidence [40] had a positive relationship with resilience. They believed that these traits made employees more resilient and motivated, and therefore, they would be better able to cope with challenges. In a review conducted in 2017, insufficient professional skills and knowledge were among the risk factors affecting the resilience of nurses [41].

The second dimension of this questionnaire was emotional-cognitive characteristics. The study results indicated that personal and contextual factors were involved in resilience. Personal factors encompassed personal characteristics, such as work-life balance, happiness, and relaxation, as well as professional characteristics, such as continuous education. Other factors are spouse or family support and clinical supervision [42]. McGee (2006) [43] mentioned some influential variables, such as self-confidence, self-discipline, flexibility, problem-solving ability, and emotional endurance. Another study noted purposefulness, faith/belief, empathy, insight, and self-care as personal characteristics [37]. Hartmann (2020) found that positive emotions played important roles in enhancing individuals’ resilience both at the individual and team levels [44]. Self-efficacy and coping skills were other important components related to resilience mentioned in many studies [45, 46].

The external support was the third dimension of the questionnaire. In a review by Çam and Büyükbayram (2017) on nurses’ resilience and its determinants, support from colleagues and the team was a protective factor affecting nurses’ resilience [41]. Other researchers have addressed personal and external factors and/or strategies that could help develop a nurse’s resilience [33, 45]. In this regard, support and resources of the workplace, such as clinical supervision, group building, and training programs for strengthening clinical performance, resilience, and well-being, have been considered group/organizational strategies that can help develop and maintain nurses’ resilience [33, 45, 46, 47]. Furthermore, cooperation in the medical team is a significant help in reducing stress and effective response [15]. Social support provides an opportunity to talk about work and stressful workflow experiences [48] and debriefing after experiencing workplace challenges [33].

Another dimension of the designed tool was behavioral strategies. Appropriate work-life balance by providing the opportunity to relax and build social relationships [39, 49, 50]. In addition, elevating recreational activities, communication with friends, enjoyable entertainment, spiritual activities, and living in the present moment [50] reinforce and enhance resilience. Happell (2013) mentioned adaptive coping strategies, such as exercise, home activities, family activities, nurses’ membership of virtual social networks, and the social clubs of staff [51].

The fifth dimension of the questionnaire was cognitive strategies. According to the reference, there should be at least three items in each factor, except for the items that have a factor loading of higher than 0.7 [28]. Given that there were two items with factor loadings of 0.92 and 0.87 in factor 5, it was maintained as a factor. Manchini and Bonano (2009) explained that although resilient people experience perturbation related to the loss or grievous events, their overall level of functioning is preserved. Furthermore, they showed that one of the beneficial components of resilience to loss is a positive worldview and beliefs in the world’s justice and more accepting attitudes toward death [52].

The reliability test of the tool revealed internal consistency. The scale’s Cronbach’s alpha was 0.915, consistent with the results of the validity assessment reported by Connor and Davidson, reporting the Cronbach’s alpha coefficient of 0.89 for the general scale of resilience [36]. Moreover, ICC had a good value of 0.888 [53]. The most common indicators of GFI were also examined, and GFI was appropriate for all indicators. Therefore, the questionnaire with five dimensions and 37 items is a suitable tool with acceptable validity and reliability for measuring nurses’ professional resilience.

### Limitations and strengths

The strength of this instrument is its specific design to assess the emergency nurses’ professional resilience. There was acceptable population diversity because the questionnaires were collected from different cities of Iran for the psychometric evaluation of the instrument. The CFA was also performed on a sample rather than the primary sample, and the results indicated the suitability of the instrument. The non-determination of cut-off points was the weakness of the instrument. Moreover, this research was only conducted in Iran, and it is better to include other countries and cultures to prove its validity and reliability because cultural factors affect resilience.

## Conclusion

Emergency nursing can create emotional challenges for many nurses due to its unpredictability and stress. Nurses need to develop resilience to cope with professional problems and maintain their mental health. A special tool is needed to measure the emergency nurses’ professional resilience. Consequently, we designed and developed a new scale using multifaceted variables and operationalized them on the validated scale. The final scale consisted of 37 items and five factors that explained 75.59% of the total variance. Healthcare managers could use this scale to evaluate nurses’ professional resilience and designate them as nurses working in emergency and disaster situations.

## Supporting information

S1 Data (SAV)

S2 Data (SAV)
